# DeePathNet: A Transformer-Based Deep Learning Model Integrating Multiomic Data with Cancer Pathways

**DOI:** 10.1158/2767-9764.CRC-24-0285

**Published:** 2024-12-18

**Authors:** Zhaoxiang Cai, Rebecca C. Poulos, Adel Aref, Phillip J. Robinson, Roger R. Reddel, Qing Zhong

**Affiliations:** ProCan, Children’s Medical Research Institute, Faculty of Medicine and Health, The University of Sydney, Westmead, Australia.; ProCan, Children’s Medical Research Institute, Faculty of Medicine and Health, The University of Sydney, Westmead, Australia.; ProCan, Children’s Medical Research Institute, Faculty of Medicine and Health, The University of Sydney, Westmead, Australia.; ProCan, Children’s Medical Research Institute, Faculty of Medicine and Health, The University of Sydney, Westmead, Australia.; ProCan, Children’s Medical Research Institute, Faculty of Medicine and Health, The University of Sydney, Westmead, Australia.; ProCan, Children’s Medical Research Institute, Faculty of Medicine and Health, The University of Sydney, Westmead, Australia.

## Abstract

Multiomic data analysis incorporating machine learning has the potential to significantly improve cancer diagnosis and prognosis. Traditional machine learning methods are usually limited to omic measurements, omitting existing domain knowledge, such as the biological networks that link molecular entities in various omic data types. Here, we develop a transformer-based explainable deep learning model, DeePathNet, which integrates cancer-specific pathway information into multiomic data analysis. Using a variety of big datasets, including ProCan-DepMapSanger, Cancer Cell Line Encyclopedia, and The Cancer Genome Atlas, we demonstrate and validate that DeePathNet outperforms traditional methods for predicting drug response and classifying cancer type and subtype. Combining biomedical knowledge and state-of-the-art deep learning methods, DeePathNet enables biomarker discovery at the pathway level, maximizing the power of data-driven approaches to cancer research. DeePathNet is available on GitHub at https://github.com/CMRI-ProCan/DeePathNet.

**Significance:**

DeePathNet integrates cancer-specific biological pathways using transformer-based deep learning for enhanced cancer analysis. It outperforms existing models in predicting drug responses, cancer types, and subtypes. By enabling pathway-level biomarker discovery, DeePathNet represents a significant advancement in cancer research and could lead to more effective treatments.

## Introduction

Multiomic analysis of diverse data types enables researchers to gain insights into tumor biology and identify new and robust therapeutic targets (1). One major goal of multiomic analysis by machine learning is to predict the cancer treatment strategies that are best suited to individuals in the context of precision medicine. A variety of multiomic studies have led to the improved detection of intratumor heterogeneity, identification of novel therapeutic targets, and more robust diagnostic and predictive markers (2–5). Many of these discoveries would not have been possible by analyzing any single omic data type alone. However, performing multiomic analysis presents computational challenges due to the large amounts of data generated by high-throughput instruments and the limitations of existing multiomic data integrative methods (6, 7).

To address this, a plethora of machine learning methods have been developed for integrating large-scale multiomic data (2–4, 7–11). For example, moCluster (8) integrates multiomic data based on joint latent variable models, showing performance superior to previous methods such as iCluster (9) and iCluster Bayes (10). Likewise, mixOmics (11) provides various options for multiomic data integration, aiming to find common information among different omic data types. Variational autoencoder–based methods have also been applied to multiomic integration (bioRxiv 2024.06.26.600742; refs. 12, 13). For instance, scVAEIT (12) performs robust probabilistic modeling for single-cell multimodal data integration and imputation, and MOVE (13) utilized generative deep learning models to discover drug–omics associations in type 2 diabetes. However, these models primarily focus on learning data-driven representations and do not consider existing biomedical knowledge that links different omic data types together, such as regulatory networks.

Deep learning has been extensively applied to similar tasks in cancer research, demonstrating improved predictive performance and the ability to capture complex patterns in high-dimensional data. For instance, deep learning models have been used to integrate multiomic data for predicting survival in patients with liver cancer (14), extract biologically relevant latent spaces from cancer transcriptomes (15), and combine histologic images with genomic data to predict cancer outcomes (16). Although these methods highlight the potential of deep learning in cancer research, they primarily rely on data-driven approaches and do not incorporate existing biomedical knowledge, such as pathways or regulatory networks. Regulatory networks exist in cells to control the expression levels of various gene products through collections of functionally interacting protein or RNA macromolecules (17). However, models that incorporate existing biomedical knowledge in addition to computational inference have the potential to better capture the interactions that drive biomarker associations and to increase the predictive power and modeling capacity of these algorithms (7).

In omic studies, most existing attempts to incorporate biomedical knowledge into machine learning are limited to one single omic data type (18–21). ATHENA (22) and PARADIGM (23) support multiomic data, but they are based on linear models that are not complex enough to model the relationships among pathways. Using deep learning, several studies have attempted to incorporate existing biomedical knowledge into multiomic models (3, 24). DCell (25) and DrugCell (26) combine the neural network architecture with known gene ontology information, but they only support using gene deletions or mutation as the input. EMOGI (27) was designed based on graph neural networks (GNN; refs. 28, 29), and it integrates protein–protein interaction networks with multiomic data to predict cancer genes. However, its network architecture cannot be easily generalized to other tasks. Furthermore, a GNN-based model was developed to classify breast cancer subtypes using multiomic data, but the model is not interpretable for discovering potential new biological mechanisms or drug targets (30). Similarly, pathway models have been utilized extensively in cancer research to enhance the understanding of tumor biology and improve predictive modeling. For example, methods like PARADIGM (23) and NetGSA (31) integrate pathway information to infer pathway activities from genomic data and perform differential pathway analysis. Although these approaches leverage pathway knowledge, a cancer-specific pathway model has not yet been incorporated into the design of a deep learning model for multiomic data integration. Integrating cancer pathways (32) into multi-omic data analysis by deep learning for general tasks, such as drug response prediction and cancer type or subtype classification, remains an open research topic.

Inspired by the computational foundation of generative pretrained transformer (hayate-lab.com 2018; https://hayate-lab.com/wp-content/uploads/2023/05/43372bfa750340059ad87ac8e538c53b.pdf) that significantly contributed to the recent achievements of ChatGPT (33), we developed DeePathNet, a transformer-based (34) explainable deep learning method that integrates multiomic data, such as genomic mutation, copy number variation, gene expression, DNA methylation, protein intensity, and CRISPR–Cas9 data, among others, with knowledge of cancer pathways. The transformer architecture has enabled many breakthroughs in artificial intelligence (35–37). In molecular biology, the transformer has been used to model DNA sequence, protein tertiary structures, and drug chemical structure data (bioRxiv 2023.01.11.523679; refs. 38, 39). Furthermore, the transformer-based models have been used together with GNN to analyze mRNA and miRNA data in cancer, but these models are not interpretable (40, 41). Therefore, whether the transformer can be combined with cancer pathway information to integrate any number of omic data layers for multiomic cancer data analysis is unknown.

Our contributions in this article can be summarized as follows:

1. A novel deep learning model called DeePathNet has been developed for analyzing cancer molecular data. The novelty of DeePathNet lies in its unique design, which enables the transformer to solve previously unanswered questions in cancer research. This offers a fresh avenue for employing transformer architecture in the analysis of multiomic data within the field of cancer. By combining the transformer with domain-specific knowledge of cancer pathways, DeePathNet not only surpasses the predictive accuracy of conventional machine learning approaches but also provides reliable model explanations.
2. The performance of DeePathNet has been evaluated and validated using large-scale datasets and multiple evaluation metrics, which are not commonly included in similar studies. Exemplary tasks, including drug response prediction and cancer type and subtype classification, have been selected due to the availability of relatively large amounts of data. DeePathNet can be extended to other tasks by adjusting the number of output neurons.
3. The top omic features and cancer pathways have been reported using DeePathNet’s feature importance, facilitating the discovery of potential new biomarkers. This information provides valuable insights into the underlying mechanisms of cancer development and progression.

## Materials and Methods

### Multiomic and drug response data collection

For drug response prediction, multiomic data were retrieved from 941 Cell Lines Project (CLP; ref. 42) and 696 Cancer Cell Line Encyclopedia (CCLE) cell lines (43). In total, 19,099 gene mutations, 19,116 copy number variations (CNV), and 15,320 gene expression features are in the CLP, and 18,103 gene mutations, 27,562 CNV, and 19,177 gene expression features are in the CCLE.

For drug response prediction analysis with proteomic data, the ProCan-DepMapSanger dataset (44) was added to the CLP (CLP + ProCan-DepMapSanger = CLP^+^) and the CCLE’s proteomic dataset (45) was also used (CCLE + CCLE proteomic data = CCLE^+^). The ProCan-DepMapSanger and CCLE proteomic datasets contain 8,498 and 12,755 protein features, respectively. The combined datasets have 910 and 292 cell lines for CLP^+^ and CCLE^+^, respectively. No additional processing was performed on the datasets (Supplementary Table S1). When using the CCLE and CCLE^+^ as the independent test set, we excluded the cell lines that are also in the CLP dataset, and only used the 71 and 33 cell lines that are unique in the CCLE and CCLE^+^ datasets, respectively.

For cancer type and subtype classification, multiomic data from The Cancer Genome Atlas (TCGA) cohorts were retrieved using TCGA-assembler 2 (46). In total, 6,356 samples were collected, containing 31,949 features from gene mutation, 23,529 features from CNV and 20,435 features from gene expression. In addition, multiomic data from 122 breast cancer samples were retrieved from a Clinical Proteomic Tumor Analysis Consortium (CPTAC) breast cancer cohort (47), containing 11,877 features from gene mutation, 23,692 features from CNV, and 23,121 features from gene expression. For breast cancer subtype classification, the Prediction Analysis of Microarray 50 (PAM50) classification (luminal A, luminal B, HER2^+^, basal, and normal-like) was retrieved from the TCGA and CPTAC datasets (Supplementary Table S1).

When certain data modalities (e.g., gene expression) are not available for a particular gene, we represent the missing values by inserting zeros into the input vector. This approach effectively excludes the corresponding neurons from influencing the model’s predictions during training, as zero inputs do not contribute to the activation of neurons. Consequently, these neurons are ignored during weight updates, allowing the model to handle missing data without the need for imputation or exclusion of the entire sample.

### Overview of DeePathNet

**Figure 1:**
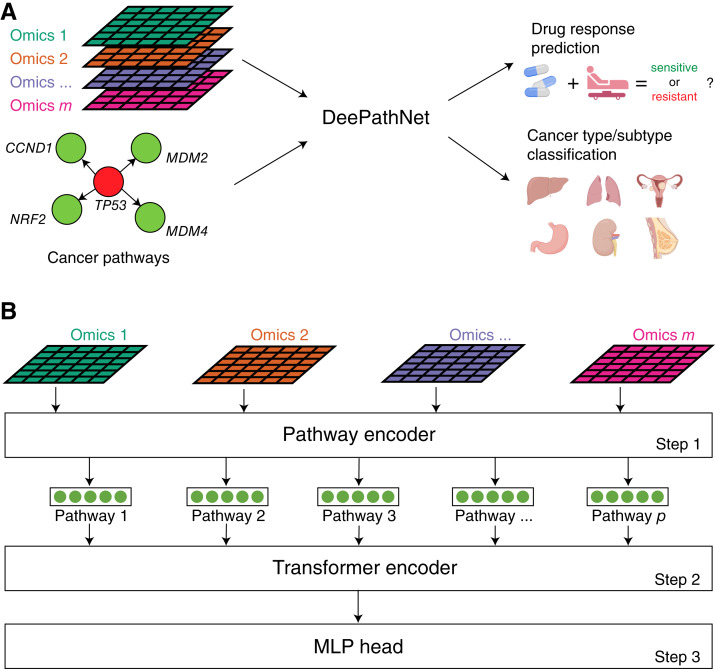
Overview of DeePathNet. **A,** DeePathNet has its network architecture built using the LCPathway dataset and takes multiomic data as the input to model pathway interactions and predict drug responses or classify cancer types and subtypes. **B,** DeePathNet architecture supports any number of omic data types as the input. Step 1: DeePathNet encodes multiomic information into cancer pathways. Step 2: DeePathNet uses a transformer encoder to learn the interactions among these pathways. Step 3: The encoded pathway vector is passed into an MLP for the prediction. Circles represent neurons in a neural network. Arrows represent the direction of information flow. (**A,** Created with BioRender.com.)

DeePathNet was developed to model biological pathways using a transformer-based deep learning architecture with both multiomic data and cancer pathway information as the input (Fig. 1A). The performance of DeePathNet was evaluated on drug response prediction and cancer type and subtype classification.

DeePathNet consists of three major steps. It starts with a pathway encoder to summarize features from omic data types into cancer pathways (step 1; Fig. 1B), and then uses a transformer encoder to model the interactions among these pathways (step 2). This is followed by a multi-layer perceptron (MLP) that can be adapted to different prediction tasks (step 3).

In step 1, the neural network architecture is constructed based on the LCPathways dataset (32), which contains 241 literature-curated pathways encompassing 3,164 cancer genes. The LCPathways dataset was selected because it is one of the most recent and comprehensive pathway databases that are specifically curated for cancer research. As such, it is particularly suitable for the applications of DeePathNet. The pathway encoder then uses a fully connected layer to project the multiomic data (omics 1 − *m*) from genes (gene 1 − *n*) onto a 512-dimension pathway vector that represents one of the cancer pathways (pathway 1−*p*; see “Materials and Methods”; Supplementary Fig. S1A). With this architecture, the pathway encoder allows DeePathNet to capture interactions across different omic data types. Moreover, grouping multiomic features into pathways reduces the dimensionality of the original high-dimensional data. This mitigates the challenge of the curse of dimensionality, making it computationally feasible to apply the model to large datasets without sacrificing its performance (48).

In step 2, an enhanced version of the transformer module is developed to encode the interactions among cancer pathways (see “Materials and Methods”; Supplementary Fig. S1B). First, a dropout layer is used to train only half of the pathways at each iteration, which prevents the model from focusing on specific pathways that may not generalize well to a test dataset. Then, two blocks of the original transformer module (34) are used, which contains a list of recurring layers, with each layer comprising a sequence of layer normalization, multi-head self-attention, and an MLP. The transformer also enables dynamic modeling of the complex relationships among cancer pathways, thus avoiding the generation of fixed weights for the different inputs, as in traditional machine learning.

In step 3, an MLP is used to map the encoded pathway vectors to output neurons, which allows the knowledge learned by the transformer module to be adapted to general prediction tasks. The three components of DeePathNet work together and cannot be applied to multiomic data individually.

### Details of DeePathNet

DeePathNet has a pathway encoder (step 1), a transformer encoder (step 2), and a MLP (step 3). The input vector for each gene is a concatenation of features from all available data modalities. For example, if mutation, CNV, and RNA data are available, the input vector ***x_i_*** for gene *i* is constructed as $x_{i}=\left[x_{i}^{mutation},\;x_{i}^{CNV},x_{i}^{RNA}\right]$, in which each $x_{i}^{modality}$ represents the feature value for that modality. If a modality is missing for a gene, we insert a zero in place of $x_{i}^{modality}$.

In step 1, DeePathNet encodes multiomic information into cancer pathways, defined by the 241 cancer pathways in LCpathways (32). Let $g_{mutation}\in \left\{0,1\right\}$ represent the mutation, $g_{CNV}\in R$ the CNV, $g_{RNA}\in R$ the gene expression, and $g_{prot}\in R$ the protein intensity of a gene *g*. Then the vector that contains omic features for a pathway that contains *n* genes with four omic data types, is defined as

$$a_{omics}=\;\left[g_{mutation}^{1}{,g}_{CNV}^{1},g_{RNA}^{1},g_{prot}^{1},\;\ldots ,g_{mutation}^{n},g_{CNV}^{n},g_{RNA}^{n},g_{prot}^{n}\right]$$

Next, the vector $a_{omics}$ is encoded into the pathway vector $a_{encoded}$ via an MLP. Here, the notation is converted into the matrix form to include the number of samples. Thus, for *N* samples, the total features from the four omic data types for a pathway can be represented as a matrix $A_{omics}$ of dimension N×4n. DeePathNet then uses a fully connected layer to encode these omic features into an encoded pathway matrix $A_{encoded}$, calculated as

$$A_{encoded}=A_{omics}W^{T}+B$$

in which W and B represent the learnable weights matrix and bias term in the fully connected layer, respectively. The dimension of the weight matrix W is set as 512 × 4*n*, and the dimension of both $A_{encoded}$ and *B* is N×512. In total, 241 cancer pathways were used, and 241 matrices $A_{encoded}^{1},A_{encoded}^{2},\ldots ,A_{encoded}^{241}$ are combined as a tensor $A_{encoded}$ with a dimensionality of N×512×241. $A_{encoded}$ is used as the input into the transformer encoder (Supplementary Fig. S1).

In step 2, DeePathNet uses a transformer encoder to learn the interdependence among regulatory pathways in cancer. In contrast to the general attention mechanism that models the interdependence between the input and target, self-attention is used by the transformer module to model interdependence within the input (i.e., features from the multiomic data; ref. 49). The transformer encoder starts with a dropout layer with a probability of 0.5 on the 241 cancer pathways, ensuring that on average half of the pathways are dropped out during training to prevent potential overfitting. The set of selected pathways is sampled independently for each training batch, allowing different pathways to be used. The transformer block was configured the same way as the original version (34), denoted as Transformer below. Because the transformer encoder contains recurrent layers, we use a superscript with parenthesis to represent the $A_{encoded}$ at different layers, in which $A_{encoded}^{\left(0\right)}$ represents the data before entering the first layer. After the first layer of the transformer block, $A_{encoded}^{\left(0\right)}$ becomes $A_{encoded}^{\left(1\right)}$ as follows:

$$A_{encoded}^{\left(1\right)}=Transformer\left(A_{encoded}^{\left(0\right)}\right)$$

DeePathNet contains two layers of transformer block, therefore:

$$A_{encoded}^{\left(2\right)}=Transformer\left(A_{encoded}^{\left(1\right)}\right)$$

Finally, in step 3, DeePathNet uses a MLP to map $A_{encoded}^{\left(2\right)}$ to the final prediction. The output dimension of a MLP depends on the prediction task. For drug response prediction, the number of output dimensions is equal to the number of drugs, and for cancer type and subtype classification, the number of output dimensions is equal to the number of cancer types and subtypes.

### Model training

The random forest models were trained with *n*_estimators = 100 and Gini impurity being the split criterion. The elastic net models were trained with alpha = 1, l1_ratio = 0.5 with 1,000 iterations. For principal component analysis (PCA) models, top 200 principal components were utilized. *k*-nearest neighbors (*k*-NN) models were trained with *k* = 5. Other details, including the default settings of the other compared methods, can be found in the official API of scikit-learn (v1.0.2, RRID: SCR_002577). For mixOmics, the block.spls mode was utilized for multiomic data integration, with ncomp set to 50 for each omic datatype. For moCluster, the mbpca function was utilized, with ncomp = 200, *k* = “all,” method = “globalScore,” option = “lambda1.” As PCA, moCluster, MOVE and scVAEIT are unsupervised methods that reduce the dimensionality of the data but do not perform classification or regression tasks on their own, we used random forest as a downstream predictive model. Specifically, after applying these four methods to the multiomic data to obtain lower-dimensional representations, we trained a random forest classifier on these features to perform the supervised prediction tasks. This approach allowed us to evaluate the predictive power of the features extracted by PCA, moCluster, MOVE, and scVAEIT in combination with a robust classification algorithm. The hyperparameters used for DeePathNet can be found in the GitHub repository. To train DeePathNet for regression, mean squared error loss was computed between the predicted and actual IC_50_. For classification, we computed the cross-entropy loss to train DeePathNet.

To address the challenge posed by the limited sample size for deep learning, hyperparameter tuning was not performed for each method. Conducting a comprehensive grid search would necessitate reserving a portion of the dataset for final performance evaluation, thereby reducing the training set size and potentially compromising model performance. Given the small changes observed and the significant time required for extensive hyperparameter tuning, all methods were trained using default hyperparameters. This decision ensures a consistent comparison across different models without the added variability introduced by individualized hyperparameter optimization.

The computation time of DeePathNet is reported in Supplementary Table S2 and was only compared with random forest and elastic net.

### Ablation study details

We performed an ablation study on all three tasks, drug response prediction, cancer type classification, and breast cancer subtype classification. The study compared the performance of DeePathNet with two controls: (i) a transformer-only model in which cancer pathways were replaced by randomly selected genes, and (ii) a plain neural network without cancer pathways or transformer architecture.

#### Training and test data

For all tasks, the same datasets were used to ensure a consistent comparison. For the drug response prediction task, the CLP dataset was used for training, and the CCLE dataset served as the independent test set. For cancer type classification, multiomic data from 6,356 TCGA samples were utilized, with 5-fold cross-validation applied to split the dataset into training and test sets. Finally, for the breast cancer subtype classification task, the multiomic data from 974 breast cancer samples in the TCGA dataset were used for training, whereas the independent CPTAC cohort consisting of 122 samples was employed as the test set.

#### Creation of the transformer-only model

The transformer-only model was generated by replacing the biologically curated cancer pathways in DeePathNet with randomly selected genes. Pathways in the transformer-only model were constructed to match the size of the original pathways but without regard to biological relevance. This randomization was performed 10 times to reduce the influence of any single random configuration. The same DeePathNet architecture was applied to these random pathways, allowing for a direct comparison between the transformer-only model and biologically meaningful pathways.

#### Evaluation metrics

Performance was assessed using the same metrics for all tasks. For drug response prediction, the evaluation metrics included *R*^2^, mean absolute error (MAE), and Pearson’s correlation coefficient. For cancer type classification, and breast cancer subtype classification, metrics included accuracy, macro-average *F*_1_-score, area under the ROC curve (AUROC), and area under the precision-recall curve (AUPRC). These metrics were computed for both the independent test sets (CCLE for drug response and CPTAC for breast cancer subtypes) and for cross-validation on the TCGA dataset (for cancer type classification).

### Data availability

All data used in this study are publicly available datasets. Data can be downloaded from the original publication as cited. Specifically, https://cellmodelpassports.sanger.ac.uk/downloads for CLP and CLP^+^, https://depmap.org/portal/ for CCLE data, https://github.com/BioinformaticsFMRP/TCGAbiolinks for TCGA data, and https://proteomic.datacommons.cancer.gov/pdc/ for CPTAC data. The source code and documentation of DeePathNet are available at https://github.com/CMRI-ProCan/DeePathNet. Source code, processed datasets, and intermediate files are also available at https://doi.org/10.6084/m9.figshare.24137619.

## Results

### DeePathNet predicts drug response

We first assessed the predictive performance of DeePathNet on a regression task by benchmarking it against random forest (50), elastic net (51), PCA, mixOmics (4), moCluster (8), MOVE (13) and scVAEIT (12) to predict the responses of anticancer drugs to cancer cell lines. These eight methods were evaluated using data from the CLP (42) and the CCLE (43), the two largest publicly available multiomic cancer cell line datasets (see “Materials and Methods”; Supplementary Table S1). DCell (25), DrugCell (26), and EMOGI (27) were not included in the benchmark as they do not support multiomic data and general prediction tasks. Gene mutation, CNV, and gene expression data from the two datasets were used as the input. For drug response data, we retrieved the IC_50_ from the Genomics of Drug Sensitivity in Cancer (GDSC; ref. 42) database. For each method, six experimental setups were assessed, comprising two datasets and three evaluation metrics, namely coefficient of determination (*R*^2^), MAE, and Pearson correlation coefficient (Pearson’s *r*) between predicted and actual IC_50_ values (see Supplementary Materials and Methods).

In DeePathNet, 241 pathway encoders were constructed (Supplementary Fig. S1A) to summarize the omic data into pathway vectors defined by the LCPathways (32). These vectors were then fed into the transformer module to model the interactions among cancer pathways (Supplementary Fig. S1B). Default hyperparameters were used for all eight methods (see “Materials and Methods”). Omic data were combined using early integration (7) for random forest and elastic net. Middle integration (7) was used for PCA, moCluster, and mixOmics. PCA and moCluster were coupled with random forest for predictions (see “Materials and Methods”; ref. 7).

**Figure 2:**
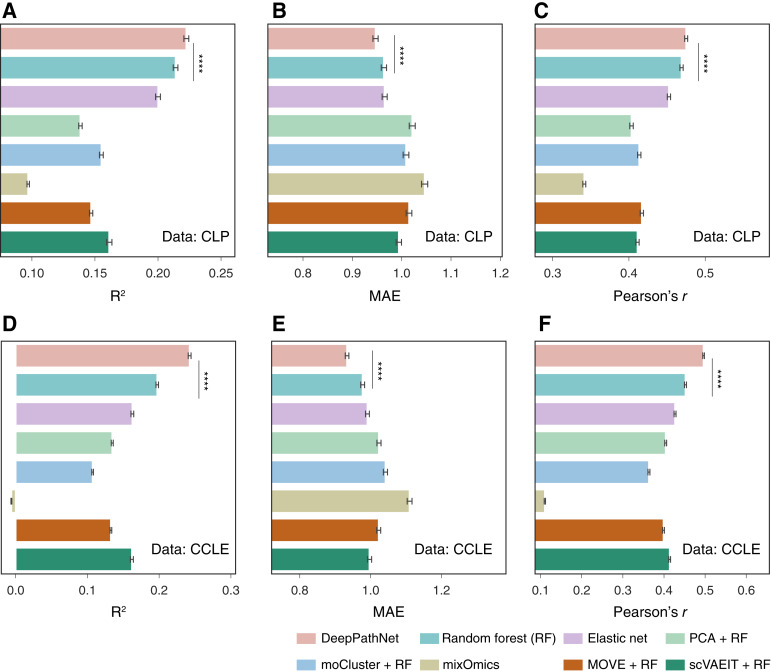
Performance evaluation of drug response prediction by cross-validation. **A–F,** Bar plots showing predictive performances across six experimental setups on the CLP and CCLE datasets by three evaluation metrics: *R*^2^ (higher indicates more accurate), MAE (lower indicates more accurate), and Pearson’s *r* (higher indicates more accurate). Error bars are derived from cross-validation, representing 95% CI of the mean. ****, *P* < 0.0001 by two-tailed paired Student *t* test, only showing significance between the first- and second-best performing methods. The eight methods are color-coded at the bottom.

To quantitatively and reliably compare the eight methods, 5-fold cross-validation was repeated five times at random, yielding 25 error measures for each of the *R*^2^, MAE, and Pearson’s *r* metrics. The mean and 95% confidence interval (CI) of the evaluation metrics was reported, serving as an estimate of the generalization error. We observed that DeePathNet had significant and consistently better performance in drug response prediction than the other seven methods that do not incorporate cancer pathway information (Fig. 2A–F; *P* value < 0.0001; two-tailed paired Student *t* test; Supplementary Table S3). By ranking the methods according to the mean measures for each setup, we found that random forest was the second-best performing method. To assess whether drug responses that were more difficult for DeePathNet to predict were similarly challenging for other methods, we calculated the correlations between DeePathNet’s predictions and those of the other seven methods. We observed that the predictive performance of DeePathNet closely aligned with that of the other seven methods we evaluated, demonstrating consistent agreement across different approaches (Pearson’s *r* > 0.9). DeePathNet showed slightly better performance overall, as reflected in its consistently higher accuracy across multiple metrics (Supplementary Fig. S2A and S2B). This concordance in performance indicates that DeePathNet does not produce any unexpected or anomalous predictions compared with these established methods. We found that the predictive performance of the paired methods was highly concordant (Pearson’s *r* > 0.9), with DeePathNet consistently outperforming the other seven methods (Supplementary Fig. S2A and S2B).

**Figure 3:**
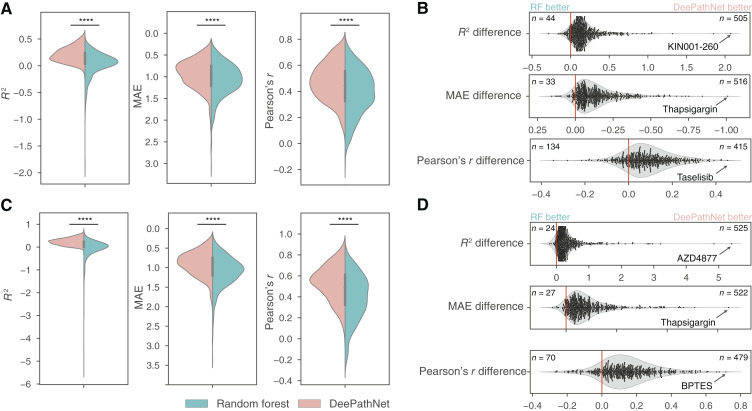
Generalization error of DeePathNet and random forest for drug response prediction. **A,** Violin plots showing predictive performances of DeePathNet and random forest using CLP as the training set and evaluated on the independent CCLE test set across the 549 GDSC drugs. The vertical axis is inverted for MAE. ****, *P* value < 0.0001 by two-tailed paired Student *t* test. **B,** Violin and swarm plots showing the performance difference in *R*^2^ (top), MAE (middle), and Pearson’s *r* (bottom) between DeePathNet and random forest for each drug. A drug is more accurately predicted by DeePathNet when it exhibits a positive value for the *R*^2^ or Pearson’s *r* difference, or a negative value of the MAE difference (the horizontal axis is inverted for MAE). The numbers of drugs more accurately predicted by DeePathNet or random forest are annotated on the top right and left of the plot, respectively. The name of the drug that achieved the largest improvement with DeePathNet is annotated for each metric. **C** and **D,** Similar to **A** and **B**, but using CLP^+^ as the training set and CCLE^+^ as the independent test set.

To validate the model’s performance using an independent test set, we trained DeePathNet on the CLP dataset (Supplementary Table S1) and tested the final model by predicting drug responses in the CCLE dataset (Supplementary Table S1). Overlapping cell lines between CLP and CCLE were excluded from the test set (see “Materials and Methods”). Cancer pathway information was integrated in the same way as described above, and a random forest model was trained as the baseline. The test performance for all 549 GDSC anticancer drugs was summarized for both DeePathNet and random forest. DeePathNet achieved statistically significantly higher predictive performance than random forest across all three metrics (Fig. 3A; *P* value < 0.0001; two-tailed paired Student *t* test; Supplementary Table S4).

To compare the predictive performance of DeePathNet with random forest for each drug, the difference of *R*^2^ between DeePathNet and random forest was measured. Here, 92% (505/549) of drugs had positive values, indicating superior predictive performance from DeePathNet over random forest. Similarly, 94% (516/549) of drugs and 76% (415/549) of drugs exhibited improved results by MAE and Pearson’s *r*, respectively (Fig. 3B). This demonstrates that DeePathNet consistently achieved better predictive performance than random forest for most anticancer drugs. The drug that obtained the largest *R*^2^ improvement by DeePathNet was KIN001-260 (Fig. 3B), which was poorly predicted by random forest and caused a long tail in the distribution of *R*^2^ values (Fig. 3A). Drugs that had the largest improvement in MAE and Pearson’s *r* with DeePathNet were thapsigargin and taselisib (Fig. 3B).

Next, we extended our analysis by including two proteomic cell line datasets from ProCan-DepMapSanger (44) and CCLE (45). ProCan-DepMapSanger is a recently published pan-cancer proteomic dataset of 949 human cell lines generated by our team, supplementing the CLP with proteomic information. DeePathNet and random forest were trained on the combined CLP and ProCan-DepMapSanger datasets (CLP^+^; Supplementary Table S1), with the final model tested on the expanded CCLE dataset that includes additional proteomic measurements (CCLE^+^; Supplementary Table S1). Pathway information was integrated into DeePathNet as described above. DeePathNet yielded significantly higher test performance than random forest across all three metrics when predicting the 549 GDSC anticancer drugs (Fig. 3C; Supplementary Table S4). Analyzing the predictive performance for each drug, DeePathNet also provided significant improvement for the majority of anticancer drugs compared with random forest (Fig. 3D). The drugs that had the largest improvement by DeePathNet were AZD4877, thapsigargin, and BPTES measured by the differences of *R*^2^, MAE, and Pearson’s *r*, respectively (Fig. 3D).

To investigate which types of drugs were most accurately predicted by DeePathNet, we grouped the 549 drugs by their canonical target cellular pathways. Drugs targeting ABL signaling and ERK MAPK signaling pathway had the highest mean Pearson’s *r* between predicted and actual IC_50_ values (Supplementary Fig. S3A). The top 20 most accurately predicted drugs and their pathways are reported in Supplementary Fig. S3B.

Taking these observations together, we demonstrated that DeePathNet increased predictive performance through several benchmarking analyses to predict responses to several drugs targeting various signaling pathways. The performance of DeePathNet on drug response prediction was also validated using independent datasets.

### DeePathNet classifies cancer types

To evaluate DeePathNet on a classification task, we used publicly available data from TCGA (52) to classify primary cancer types. Gene mutation, CNV, and gene expression features were used as the omic data input to train DeePathNet models to classify each of the 6,356 samples into one of 23 cancer types (see “Materials and Methods”). A total of seven metrics were used across the analyses to ensure reliable evaluation. These metrics are accuracy, macro-average *F*_1_-score, precision, recall (sensitivity), AUROC, AUPRC, and stability (see Supplementary Materials and Methods). LCPathways was integrated in the same way as described for the drug response prediction. For benchmarking, the elastic net was replaced by the *k*-NN (53). This was done because elastic net regularization is less commonly used for the task of cancer type classification, whereas *k*-NN is a more widely adopted method for classification. For all nine methods, feature integration and hyperparameter settings were identical to the drug response prediction.

**Figure 4:**
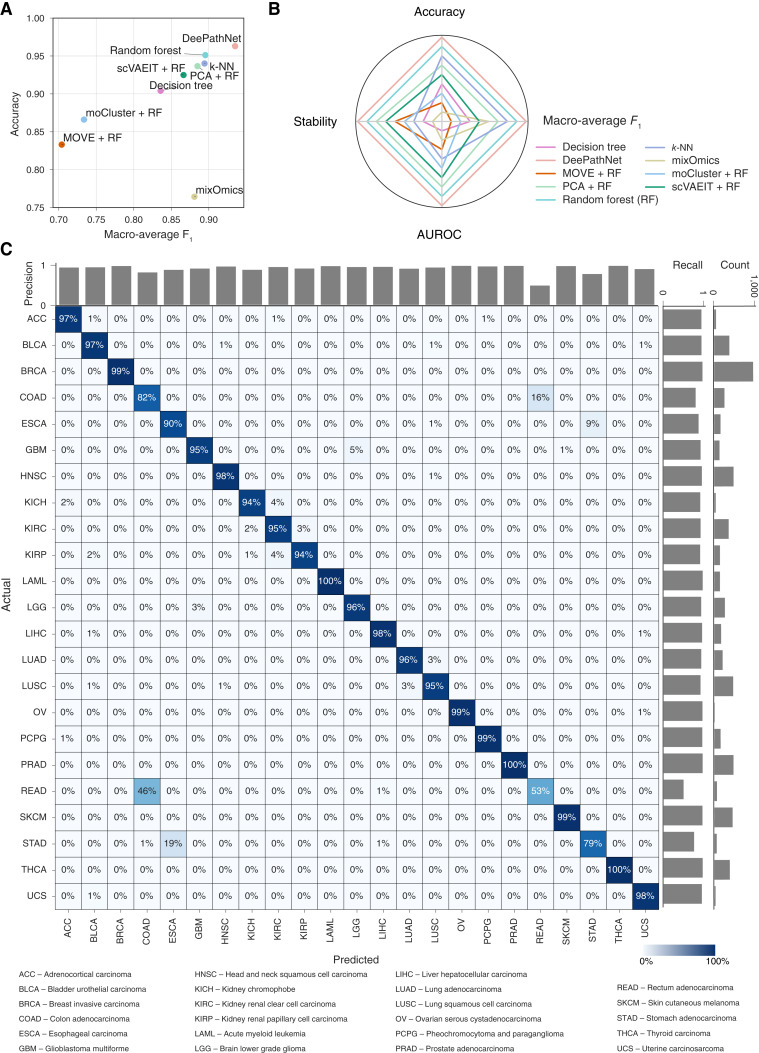
Performance evaluation of cancer type classification. **A,** Model comparison using cross-validation on the TCGA dataset. The *x*-axis represents the macro-average *F*_1_-score, and the *y*-axis denotes accuracy. **B,** Radar chart showing the model ranks across the set of four metrics. A larger enclosed area indicates better predictive performance. **C,** Confusion matrix for the classification of 23 cancer types. Columns denote predicted labels, and rows represent actual labels. The percentage shown represents the proportion of predictions made for the corresponding cancer type, with each row summing to 1. The diagonal represents correct predictions for each cancer type, with the percentage indicating the recall. Bar plots show precision (horizontal axis), recall (vertical axis, leftmost), and number of samples (vertical axis, rightmost) per cancer type.

In the absence of an independent dataset comprising the 23 cancer types, cross-validation was performed for these nine methods on the TCGA dataset, and the mean and 95% CI of the evaluation metrics were reported as an estimate of the generalization error. DeePathNet consistently outperformed the other eight machine learning methods by accuracy and macro-average *F*_1_-score (Fig. 4A; Supplementary Table S5). In contrast, other methods, such as mixOmics, only performed well in one metric, indicating that these methods may be suitable for certain scenarios but cannot generalize well across different prediction tasks (Fig. 4A). Assessing the performance of each method using a set of four metrics including accuracy, macro-average *F*_1_-score, AUROC and stability showed that DeePathNet was consistently top-ranked, followed by random forest (Fig. 4B; Supplementary Table S5).

To further investigate DeePathNet’s performance for each cancer type, the predicted and actual cancer type for each sample was visualized using a confusion matrix, with the number of samples, precision, and recall annotated (Fig. 4C). DeePathNet achieved a recall of more than 0.95 for most cancer types, with acute myeloid leukemia, pancreatic adenocarcinoma, and thyroid carcinoma as the top three most accurately classified cancer types. Rectum adenocarcinoma was the cancer type with the lowest recall, with 46% of the samples incorrectly classified as colon adenocarcinoma. The latter outcome is unsurprising because the colon and rectum are adjacent tissue types that share highly similar features, with these two cancer types often grouped together (54) and are treated with similar chemotherapeutic regimens (54). The cancer type exhibiting the second-lowest recall was stomach adenocarcinomas, with 19% of stomach adenocarcinoma samples incorrectly classified as esophageal carcinoma. This can be explained by their similar histopathology and the anatomical proximity of stomach adenocarcinoma and esophageal carcinoma (55). Next, AUROC and AUPRC were examined for each cancer type, both displaying high performances for all cancer types, with the exception of AUPRC for rectum adenocarcinoma, due to the tissue proximity of rectum adenocarcinoma to colon adenocarcinoma (Supplementary Fig. S4A and S4B).

### DeePathNet classifies breast cancer subtypes

Gene mutation, CNV, and gene expression features were used to train DeePathNet models for the classification of five breast cancer subtypes (luminal A, luminal B, HER2^+^, basal, and normal-like) according to the PAM50 test (56). A total of 974 breast cancer samples from the TCGA dataset were used for training, and a breast cancer cohort of 122 samples from the CPTAC was included as an independent dataset to evaluate the generalization error.

**Figure 5:**
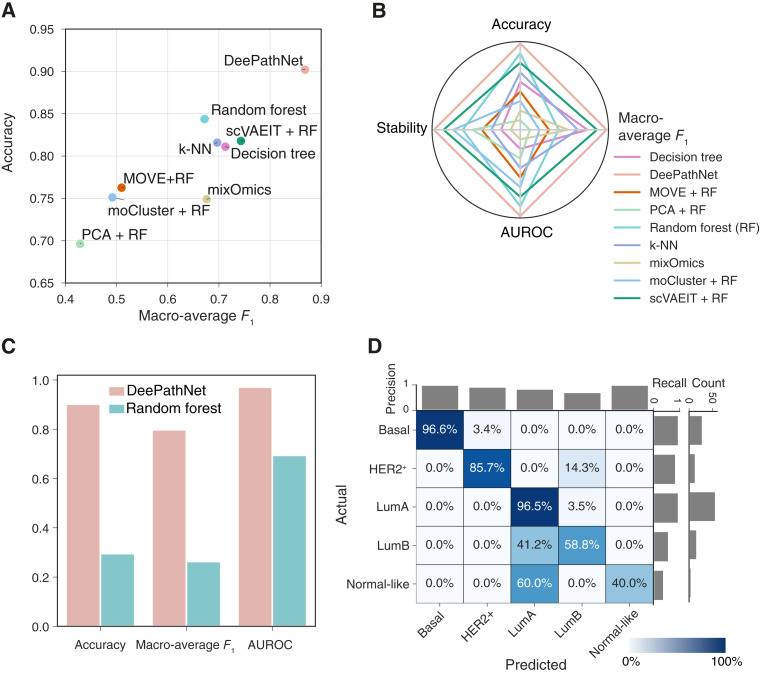
Performance evaluation of breast cancer subtype classification. **A,** Model evaluation by cross-validation. The *x*-axis represents the macro-average *F*_1_-score, and the *y*-axis represents accuracy. **B,** Radar chart showing the model ranks across the set of four metrics. A larger enclosed area represents better classification performance. **C,** Performance metrics showing generalization errors for DeePathNet and random forest when using CPTAC data as the independent test set for validation. **D,** Confusion matrix showing generalization errors when using CPTAC data as the independent test set for validation. Statistics are annotated in the same way as described in Fig. 4C.

Cross-validation for all nine methods was first performed on the TCGA dataset, reporting the mean and 95% CI of the evaluation metrics as an estimate of the generalization error. DeePathNet substantially improved over the other methods in terms of accuracy and macro-average *F*_1_-score (Fig. 5A; Supplementary Table S6). The performance gain in AUROC was relatively minor but statistically significant (Student *t* test *P* value < 5 × 10^−4^; Supplementary Table S6). The methods were then ranked according to the same set of four metrics as in cancer type classification. DeePathNet achieved the best performance in all four metrics, with random forest ranked as the second best overall (Fig. 5B). Other methods showed inconsistent performance rankings across different metrics, demonstrating the necessity of using multiple evaluation metrics for a comprehensive evaluation.

To validate the model’s performance on an independent test set, a DeePathNet model was trained on the TCGA breast cancer cohort, with the final model tested on the independent CPTAC breast cancer cohort. Benchmarked against random forest, DeePathNet yielded a much lower generalization error on the independent test set (Fig. 5C; Supplementary Table S7). Next, the generalization error of DeePathNet was assessed for each subtype by a confusion matrix. DeePathNet achieved the highest precision and recall in classifying the basal subtype (96.6%; Fig. 5D), with most tumors in this subtype being high-grade with a poor prognosis (57). The most difficult subtype to classify was normal-like, in which three out of the five normal-like samples were incorrectly classified as luminal A (Fig. 5D). Luminal A and normal-like subtypes are traditionally difficult to distinguish as they share the same IHC markers (57). The normal-like subtype is less frequently used in clinics (58). Further analyses by AUROC (Supplementary Fig. S5A) and AUPRC (Supplementary Fig. S5B) demonstrated DeePathNet’s high predictive performance for each subtype. Overall, these results demonstrated the performance of DeePathNet on cancer subtype classification using both cross-validation and validation using an independent dataset.

### DeePathNet provides model explanations

The DeePathNet model is explainable at both omic and pathway levels by using feature importance derived from SHapley Additive exPlanations (SHAP; ref. 59) and Layer-wise Relevance Propagation (LRP; ref. 60). SHAP attributes the prediction to all features, assigning each feature an importance value. At the same time, LRP assumes that the classifier can be decomposed into several layers of computation, with these layers being parts of the feature extraction. Thus, SHAP and LRP are *post hoc* model explanation approaches that establish relationships between feature values and the predictions after DeePathNet is trained. Breast cancer subtype classification was used to demonstrate the model explanation.

**Figure 6:**
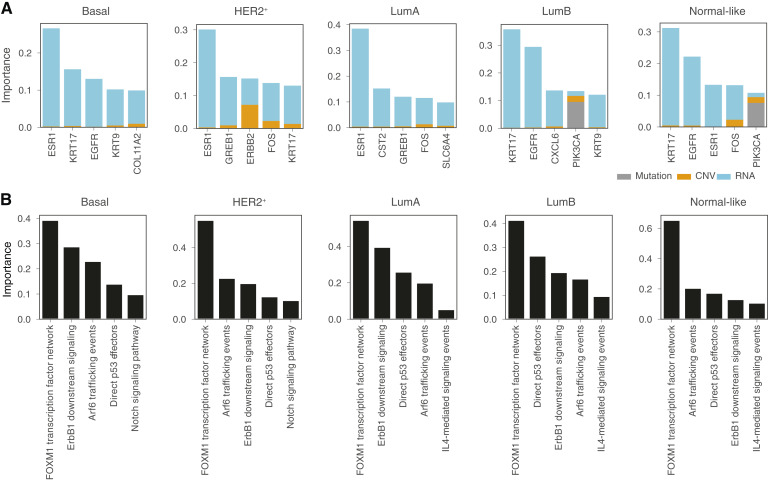
DeePathNet model explanation by omic level and pathway level feature importance. **A,** Stacked bar plots showing the omic level feature importance of the top five genes for each omic data type (indicated by gray, yellow, and blue color). **B,** Bar plots showing the DeePathNet pathway level feature importance of the top five pathways.

To explain the model at the omic level, SHAP was used to calculate feature importance. Specifically, feature importance was computed and visualized for the top five genes as stack bar plots comprising each omic data type for each breast subtype (Fig. 6A). DeePathNet was able to identify known biomarker genes as top features, such as ESR1, ERBB2, and KRT17, whose gene expression is routinely used to determine the PAM50 subtypes in the clinic (Fig. 6A; ref. 56). Most genes had their high feature importance attributable to transcriptomic data (Fig. 6A), consistent with the fact that PAM50 classifications are RNA-based subtypes (56).

To explain the models at the pathway level, LRP was used to calculate feature importance. Because the cancer pathways are represented as an encoded vector that summarizes multiomic information, the feature importance of a cancer pathway is computed for all omic data types jointly. For each cancer subtype, the top five pathways with the highest feature importance values were ranked (Fig. 6B). DeePathNet identified the FOXM1 transcription factor network as the most important pathway for predicting all PAM50 subtypes (Fig. 6B). FOXM1 shows distinct patterns of expression in different breast cancer subtypes and is seen as a promising candidate target in breast cancer treatment (61). FOXM1 is also an adverse prognostic factor of survival in luminal A and B subtypes (62). The ARF6 pathway was shown to be overexpressed in triple-negative breast cancer and to be associated with breast cancer invasion and metastasis (63). Similarly, Notch signaling pathways are involved in cell proliferation, apoptosis, hypoxia, and epithelial-to-mesenchymal transition and were found to be overexpressed in HER2-positive and triple-negative breast cancer (64).

Taken together, these findings suggest that DeePathNet provides reliable model explanations with a strong biological basis by providing feature importance at both the omic level and pathway level.

### Ablation study

**Figure 7:**
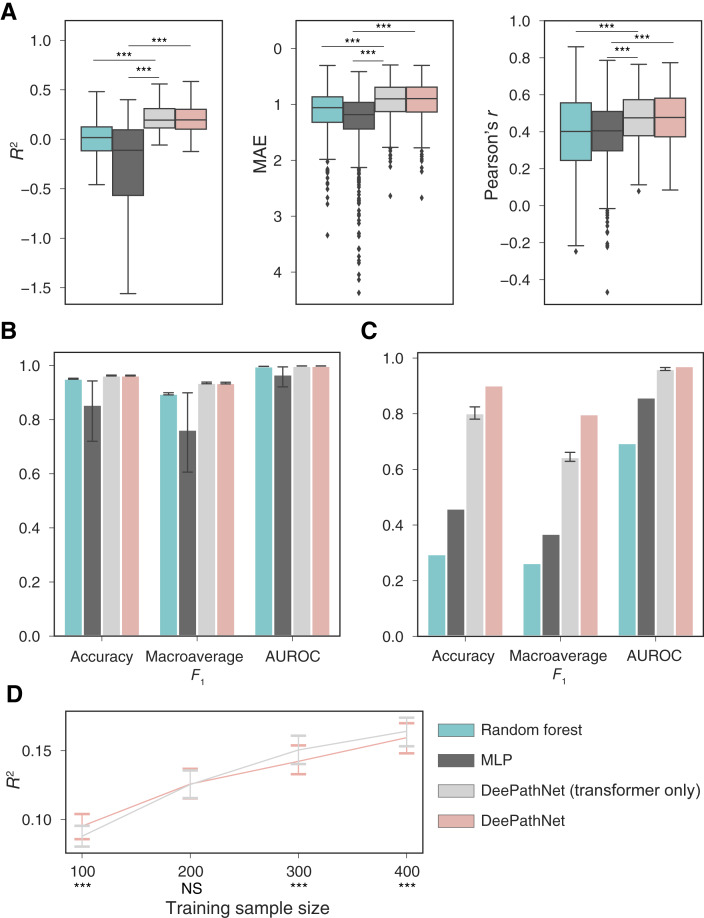
Ablation study of DeePathNet using transformer-only model and plain neural networks (MLP). **A,** Box plots showing the predictive performance from the independent test set across the 549 drugs for random forest (green), MLP (dark gray), DeePathNet with randomly wired pathways (light gray), and DeePathNet (pink). Outliers for *R*^2^ are hidden for clearer visualization. **B,** Bar plots showing predictive performances of the four models from cross-validation on cancer type classification. **C,** Bar plots showing predictive performance from the independent test set on breast cancer subtype classification. CIs represent 95% CI of the mean (*n* = 10 experiments). **D,** Down-sample analysis showing DeePathNet with cancer pathways performs better on drug response prediction with smaller training sample sizes compared with DeePathNet with random wired pathways. The solid line represents the mean *R*^2^, and CIs represent 95% CIs of the mean (*n* = 10 experiments). ***, *P* < 0.001, paired *t* test.

Lastly, we assessed the predictive capability of DeePathNet by separately examining its two primary components: the pathway encoder and the transformer module. To evaluate the additional predictive strength by integrating cancer pathway knowledge, we devised a control by randomly altering the pathway definitions for DeePathNet. This process was repeated 10 fold. Compared with the transformer-only model, DeePathNet provided more accurate predictions across all the metrics for breast cancer subtype classification, whereas similar outcomes were observed for drug response prediction and cancer type classification (Fig. 7A–C; Supplementary Tables S8 and S9). Such comparable performance between randomly configured and manually designed neural networks was also observed previously (26, 65). Without losing any predictive power, neural networks designed using human knowledge have been found to provide more meaningful model explanations that could lead to biological discoveries (26, 65). Further investigation replicated the phenomenon observed in the previous study (65) demonstrating that the neural network, constructed using known biological knowledge, outperforms transformer-only model when the sample size is limited (Fig. 7D). Next, we benchmarked DeePathNet using a MLP as a plain neural network that does not use either the pathway information or the transformer. When equipped with both the pathway information and the transformer module, DeePathNet achieved significantly more accurate predictions across all considered metrics and tasks (Fig. 7A–C; Supplementary Tables S8 and S9).

In summary, DeePathNet, when integrated with the transformer module – irrespective of the presence of pathway knowledge – provided significantly higher predictive power compared with plain neural networks and random forest. The inclusion of pathway knowledge enhanced DeePathNet’s accuracy in breast cancer subtype classification and facilitated pathway level model explanation.

## Discussion

DeePathNet broadens the application of the transformer to cancer multiomic data integration by incorporating cancer pathways, which successfully overcomes the limitations of most existing machine learning approaches that do not consider known cancer biology. DeePathNet integrates multiomic data with cancer pathway knowledge to accurately predict drug responses and classify cancer types and subtypes. The self-attention mechanism of the transformer module dynamically models the interdependency among pathways, thus capturing regulatory effects across different biological processes and the effects of dysregulation. Unlike traditional machine learning models in which the influence of each input feature is fixed after training, the self-attention mechanism enables the model to assign varying levels of importance to different pathways for each individual sample during inference. This dynamic weighting allows for a more nuanced modeling of pathway interactions tailored to the specific context of each input sample. By modeling pathways instead of multiomic features directly, DeePathNet also alleviates the challenge of the curse of dimensionality (48) commonly encountered in multiomic data analysis.

The predictive performance of DeePathNet was evaluated and validated by one regression and two classification tasks. The evaluation was conducted on a larger scale than previous similar studies (8, 11) using multiple big datasets and a range of metrics with both cross-validation and independent testing. Based on the transformer architecture, DeePathNet outperformed other machine learning methods that do not incorporate pathway information and instead rely only on omic data for input features. A low generalization error when validating DeePathNet models on independent datasets suggests that DeePathNet works well even when different experimental protocols were implemented among these independent datasets. DeePathNet provides model explanations at the pathway level, which has not yet been accomplished by any other multiomic integration tools that can predict drug response or classify cancer type and subtype. DeePathNet was able to highlight known biomarkers when predicting breast cancer subtypes, including ESR1, ERBB2, and the FOXM1 network pathways. This suggests that other top-ranked features, such as ERBB1 downstream signaling pathway, may provide novel insights into cancer biology and drug discovery. The ERBB1 downstream signaling pathway, activated by EGFR, regulates key processes such as cell growth, survival, and differentiation, and further investigation may uncover new therapeutic targets or mechanisms contributing to drug resistance.

In our ablation study, we observed that although the transformer-only model achieved similar predictive performance to DeePathNet, the inclusion of pathway information offers significant advantages. Specifically, the transformer module improves accuracy by effectively modeling complex relationships among features through its self-attention mechanism. By incorporating pathway information, DeePathNet not only maintains this high level of predictive performance but also provides pathway-level feature importance, enabling us to identify the most important cancer pathways associated with the predictive outcomes. Furthermore, the incorporation of prior biological knowledge is particularly beneficial in data-sparse contexts, in which it can guide the model and enhance accuracy. In contrast, in data-rich environments, the model may learn these relationships independently, reducing the direct impact of predefined pathway knowledge on predictive performance.

Despite these comprehensive evaluations, DeePathNet has only been designed to support gene-based features at this stage. Other omic data types such as phosphoproteomics, metabolomics, and images cannot be directly utilized as the input to the pathway encoder. Therefore, future work of DeePathNet includes extending the model to support any data modalities, which can be potentially done by mapping the features into the pathway space using different types of encoders. Furthermore, a more extensive hyperparameter tuning for each of the benchmarked methods could provide a more reliable comparison and potentially improve their performance in relation to DeePathNet. The interpretability analyses demonstrated in breast cancer subtype classification can be extended to other prediction tasks, such as drug response prediction and cancer type classification, to further showcase DeePathNet’s explainability and its potential to uncover meaningful biological insights across diverse applications. Meanwhile, as large proteomic and metabolomic datasets become increasingly available, the predictive power of DeePathNet will improve because deep learning will obtain a performance boost with more data (66).

In conclusion, DeePathNet combines multiomics, deep learning, and existing biological knowledge to predict cancer phenotypes accurately with a model explanation. The application of DeePathNet may lead to more accurate diagnosis and prognosis and will facilitate researchers to understand unknown cancer mechanisms and prioritize putative drug targets.

## Supplementary Material

Figure S1 Details of pathway encoder and Transformer encoder.

Figure S2 Consistency of drug response predictions from different machine learning models

Figure S3 Analysis of performance of drug response prediction by target pathways

Figure S4 ROC curves and precision-recall curves for TCGA cancer type classification

Figure S5 ROC curves and precision-recall curves for breast cancer subtype classification

Table S1 Overview of datasets used in this study

Table S2 Computation time for DeePathNet

Table S3 Benchmarking results for drug response prediction with cross-validation

Table S4 Generalization errors for drug response prediction

Table S5 Benchmarking results for cancer type classification with cross-validation

Table S6 Benchmarking results for breast cancer subtype classification with cross-validation

Table S7 Generalization errors for breast cancer subtype classification

Table S8 Ablation study of DeePathNet on drug response prediction

Table S9 Ablation study of DeePathNet on cancer type and breast cancer subtype classification

Materials and Methods Evaluation metrics
