# Supplementary material for: DeePathNet: A Transformer-Based Deep Learning Model Integrating Multiomic Data with Cancer Pathways
Source: Cancer Res Commun. 2024 Dec 18;4(12):3151–64. doi: 10.1158/2767-9764.CRC-24-0285 (PMC11652962; doi:10.1158/2767-9764.CRC-24-0285)
Supplement: Figure S1 — Details of pathway encoder and Transformer encoder. [file crc-24-0285_figure_s1_suppsf1.docx]

Figure S1 Details of pathway encoder and Transformer encoder. **A,** Detailed illustration of the pathway encoder. A fully connected layer encodes multi-omic data from genes into a pathway vector. **B,** Detailed illustration of the Transformer encoder. Pathway vectors are first fed into a dropout layer, followed by a recurrent sequence (grey box) of layer normalisation, multi-head self-attention and multi-layer perceptron (MLP). The components in the grey box recur twice in DeePathNet. Arrows represent the direction of information flow and represents matrix addition.
