# Supplementary material for: DeePathNet: A Transformer-Based Deep Learning Model Integrating Multiomic Data with Cancer Pathways
Source: Cancer Res Commun. 2024 Dec 18;4(12):3151–64. doi: 10.1158/2767-9764.CRC-24-0285 (PMC11652962; doi:10.1158/2767-9764.CRC-24-0285)
Supplement: Figure S2 — Consistency of drug response predictions from different machine learning models [file crc-24-0285_figure_s2_suppsf2.docx]

Figure S2 Consistency of drug response predictions from different machine learning models. **A,** Scatter plots showing the predictive performance of drugs (measured by R^2^ between the observed and predicted IC50 values) from DeePathNet (vertical axis) against each of the remaining five machine learning models (horizontal axis) evaluated on the CLP dataset. The diagonal red line indicates equal performance between the two models. Points above the red line represent drugs that are more accurately predicted by DeePathNet. Pearson’s *r* (R) and *p*-value (p) are annotated. **B,** Similar to **A**, but evaluated on the CCLE dataset.
