# Supplementary material for: DeePathNet: A Transformer-Based Deep Learning Model Integrating Multiomic Data with Cancer Pathways
Source: Cancer Res Commun. 2024 Dec 18;4(12):3151–64. doi: 10.1158/2767-9764.CRC-24-0285 (PMC11652962; doi:10.1158/2767-9764.CRC-24-0285)
Supplement: Figure S3 — Analysis of performance of drug response prediction by target pathways [file crc-24-0285_figure_s3_suppsf3.docx]

Figure S3 Analysis of performance of drug response prediction by target pathways. **A,** A violin plot showing the predictive performance grouped by drug canonical target pathways, ranked by the mean Pearson’s *r* of the group. **B,** Top 20 drugs ranked by Pearson’s *r*. Drugs are coloured by their canonical target pathways.
