# Supplementary material for: DeePathNet: A Transformer-Based Deep Learning Model Integrating Multiomic Data with Cancer Pathways
Source: Cancer Res Commun. 2024 Dec 18;4(12):3151–64. doi: 10.1158/2767-9764.CRC-24-0285 (PMC11652962; doi:10.1158/2767-9764.CRC-24-0285)
Supplement: Figure S4 — ROC curves and precision-recall curves for TCGA cancer type classification [file crc-24-0285_figure_s4_suppsf4.docx]

Figure S4 ROC curves and precision-recall curves for TCGA cancer type classification. **A,** ROC curves for DeePathNet classification of TCGA cancer types. Mean AUROC and standard error of the mean are annotated. **B,** Precision-recall curves for DeePathNet classification of TCGA cancer types. Mean AUPRC and standard error of the mean are annotated. Full terms of the abbreviations in **A** and **B** are listed in **Figure 4C**.
