# Supplementary material for: DeePathNet: A Transformer-Based Deep Learning Model Integrating Multiomic Data with Cancer Pathways
Source: Cancer Res Commun. 2024 Dec 18;4(12):3151–64. doi: 10.1158/2767-9764.CRC-24-0285 (PMC11652962; doi:10.1158/2767-9764.CRC-24-0285)
Supplement: Figure S5 — ROC curves and precision-recall curves for breast cancer subtype classification [file crc-24-0285_figure_s5_suppsf5.docx]

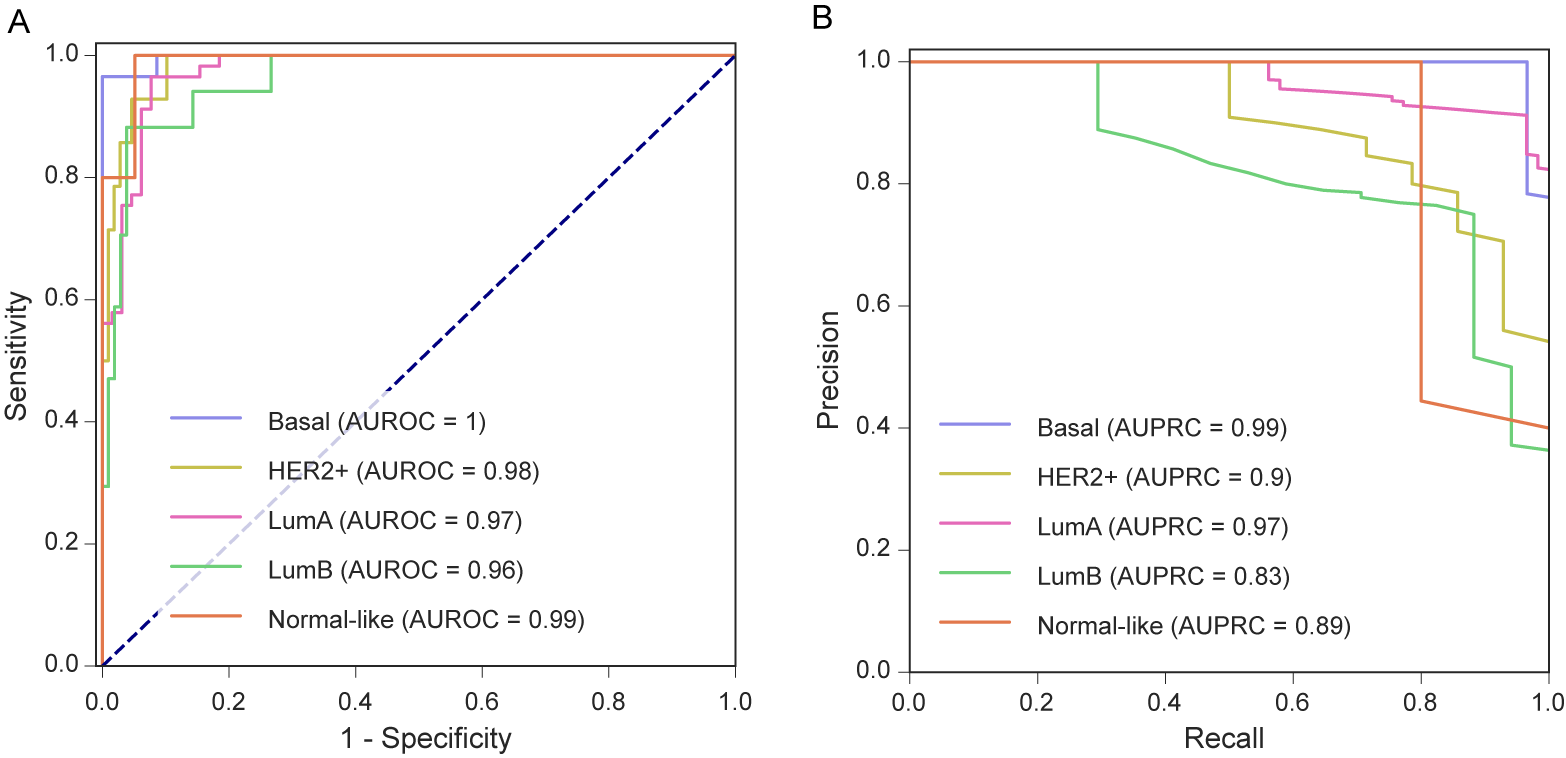


Figure S5 ROC curves and precision-recall curves for breast cancer subtype classification. **A,** ROC curves for DeePathNet classification of breast cancer subtypes using TCGA as the training data and CPTAC as the test data. **B,** Similar to **A**, but showing AUPRC.
