# Supplementary material for: DeePathNet: A Transformer-Based Deep Learning Model Integrating Multiomic Data with Cancer Pathways
Source: Cancer Res Commun. 2024 Dec 18;4(12):3151–64. doi: 10.1158/2767-9764.CRC-24-0285 (PMC11652962; doi:10.1158/2767-9764.CRC-24-0285)
Supplement: Table S1 — Overview of datasets used in this study [file crc-24-0285_table_s1_suppst1.docx]

## Table S1 Overview of datasets used in this study

| **Data sets** | **Number of features** | | | |
| --- | --- | --- | --- | --- |
| (n = sample size) | **Gene mutation** | **CNV** | **Gene expression** | **Protein** |
| **Drug response prediction (n = 549 GDSC drugs)** | | | | |
| CLP (n = 941) | 19,099 | 19,116 | 15,320 | - |
| CLP^+^ (n = 910) | 19,099 | 19,116 | 15,320 | 8,498 |
| CCLE (n = 696) | 18,103 | 27,562 | 19,117 | - |
| CCLE^+^ (n = 292) | 18,103 | 27,562 | 19,117 | 12,755 |
| **Cancer type classification** | | | | |
| TCGA (n = 6,356) | 31,949 | 23,529 | 20,435 | - |
| **Breast cancer subtype classification** | | | | |
| TCGA (n = 974) | 31,949 | 23,529 | 20,435 | - |
| CPTAC (n = 122) | 11,877 | 23,692 | 23,121 | - |

CLP^+^ = CLP + ProCan-DepMapSanger and CCLE^+^= CCLE + CCLE proteomic dataset.
