# Supplementary material for: DeePathNet: A Transformer-Based Deep Learning Model Integrating Multiomic Data with Cancer Pathways
Source: Cancer Res Commun. 2024 Dec 18;4(12):3151–64. doi: 10.1158/2767-9764.CRC-24-0285 (PMC11652962; doi:10.1158/2767-9764.CRC-24-0285)
Supplement: Table S2 — Computation time for DeePathNet [file crc-24-0285_table_s2_suppst2.docx]

## Table S2 Computation time for DeePathNet

|  | **Mean training time ± 95%CI** |  |
| --- | --- | --- |
| **DeePathNet** | 1588.32 ± 6.2 seconds |  |
| Random forest | 311.22 ± 2.3 seconds |  |
| Elastic net | 283.77 ± 2.8 seconds |  |

Time taken to train DeePathNet, random forest and elastic net for drug response prediction. The CIs were calculated from the 25 experiments as described above.
