# Supplementary material for: DeePathNet: A Transformer-Based Deep Learning Model Integrating Multiomic Data with Cancer Pathways
Source: Cancer Res Commun. 2024 Dec 18;4(12):3151–64. doi: 10.1158/2767-9764.CRC-24-0285 (PMC11652962; doi:10.1158/2767-9764.CRC-24-0285)
Supplement: Table S3 — Benchmarking results for drug response prediction with cross-validation [file crc-24-0285_table_s3_suppst3.docx]

## Table S3 Benchmarking results for drug response prediction with cross-validation

|  | **R2 mean ± 95%CI** | **MAE mean ± 95%CI** | **Pearson's r mean ± 95%CI** |
| --- | --- | --- | --- |
| **CLP** | | | |
| **DeePathNet** | **0.222 ± 0.0020** | **0.947 ± 0.0021** | **0.475 ± 0.0053** |
| Random forest (RF) | 0.214 ± 0.0018 | 0.964 ± 0.0021 | 0.469 ± 0.0054 |
| Elastic net | 0.200 ± 0.0020 | 0.965 ± 0.0023 | 0.452 ± 0.0051 |
| moCluster+RF | 0.155 ± 0.0015 | 1.009 ± 0.0022 | 0.413 ± 0.0057 |
| PCA+RF | 0.138 ± 0.0014 | 1.021 ± 0.0022 | 0.403 ± 0.0058 |
| mixOmics | 0.097 ± 0.0009 | 1.047 ± 0.0019 | 0.342 ± 0.0061 |
| MOVE+RF | 0.147 ± 0.0007 | 1.015 ± 0.0030 | 0.417 ± 0.0011 |
| scVAEIT+RF | 0.161 ± 0.0011 | 0.994 ± 0.0028 | 0.411 ± 0.0011 |
|  | | | |
| **CCLE** | | | |
| **DeePathNet** | **0.242 ± 0.0020** | **0.934 ± 0.0020** | **0.496 ± 0.0052** |
| Random forest (RF) | 0.197 ± 0.0018 | 0.977 ± 0.0022 | 0.452 ± 0.0055 |
| Elastic net | 0.163 ± 0.0019 | 0.991 ± 0.0025 | 0.427 ± 0.0053 |
| PCA+RF | 0.135 ± 0.0014 | 1.024 ± 0.0023 | 0.404 ± 0.0059 |
| moCluster+RF | 0.107 ± 0.0012 | 1.042 ± 0.0023 | 0.363 ± 0.0060 |
| mixOmics | -0.006 ± 0.0006 | 1.110 ± 0.0016 | 0.110 ± 0.0066 |
| MOVE+RF | 0.133 ± 0.0006 | 1.023 ± 0.0030 | 0.399 ± 0.0011 |
| scVAEIT+RF | 0.161 ± 0.0011 | 0.994 ± 0.0028 | 0.411 ± 0.0011 |

Benchmarking six methods to predict drug responses by reporting mean cross-validation performance with 95% confidence interval (CI). Cells in bold represent the best performance.
