# Supplementary material for: DeePathNet: A Transformer-Based Deep Learning Model Integrating Multiomic Data with Cancer Pathways
Source: Cancer Res Commun. 2024 Dec 18;4(12):3151–64. doi: 10.1158/2767-9764.CRC-24-0285 (PMC11652962; doi:10.1158/2767-9764.CRC-24-0285)
Supplement: Table S4 — Generalization errors for drug response prediction [file crc-24-0285_table_s4_suppst4.docx]

## Table S4 Generalization errors for drug response prediction

|  | **R^2^ mean ± 95%CI** | **MAE mean ± 95%CI** | **Pearson's r mean ± 95%CI** |
| --- | --- | --- | --- |
| **CLP > CCLE** | | | |
| **DeePathNet** | **0.208 ± 0.0118** | **0.933 ± 0.0274** | **0.476 ± 0.0119** |
| Random forest | 0.027 ± 0.0200 | 1.07 ± 0.0315 | 0.390 ± 0.0143 |
|  | | | |
| **CLP**^+^ **> CCLE**^+^ | | | |
| **DeePathNet** | **0.233 ± 0.0126** | **0.899 ± 0.0262** | **0.532 ± 0.0139** |
| Random forest | -0.106 ± 0.0441 | 1.107 ± 0.0330 | 0.388 ± 0.0183 |

Comparing two methods by evaluating mean generalization errors with 95% CI of drug response prediction. Cells in bold represent the best performance.
