# Supplementary material for: DeePathNet: A Transformer-Based Deep Learning Model Integrating Multiomic Data with Cancer Pathways
Source: Cancer Res Commun. 2024 Dec 18;4(12):3151–64. doi: 10.1158/2767-9764.CRC-24-0285 (PMC11652962; doi:10.1158/2767-9764.CRC-24-0285)
Supplement: Table S5 — Benchmarking results for cancer type classification with cross-validation [file crc-24-0285_table_s5_suppst5.docx]

## Table S5 Benchmarking results for cancer type classification with cross-validation

|  | **Accuracy mean**  **± 95%CI** | **Macro-average F1-score mean**  **± 95%CI** | **AUROC mean**  **± 95%CI** | **Stability** |
| --- | --- | --- | --- | --- |
| **DeePathNet** | **0.963 ± 0.0015** | **0.935 ±0.0030** | **0.998 ± 0.0001** | **0.004** |
| Random forest (RF) | 0.951 ± 0.0018 | 0.895 ± 0.0038 | 0.997 ± 0.0003 | 0.005 |
| *k*-NN | 0.940 ± 0.0018 | 0.894 ± 0.0045 | 0.982 ± 0.0016 | 0.007 |
| PCA+RF | 0.937 ± 0.0023 | 0.885 ± 0.0039 | 0.996 ± 0.0003 | 0.005 |
| moCluster | 0.866 ± 0.0031 | 0.734 ± 0.0034 | 0.987 ± 0.0010 | 0.006 |
| mixOmics+RF | 0.764 ± 0.0040 | 0.881 ± 0.0091 | 0.919 ± 0.0041 | 0.015 |
| MOVE+RF | 0.833 ± 0.0056 | 0.704 ± 0.0041 | 0.978 ± 0.0020 | 0.006 |
| scVAEIT+RF | 0.925 ± 0.0034 | 0.866 ± 0.0064 | 0.994 ± 0.0012 | 0.006 |

Benchmarking six methods to predict cancer types by reporting cross-validation performance. Cells in bold represent the best performance.
