# Supplementary material for: DeePathNet: A Transformer-Based Deep Learning Model Integrating Multiomic Data with Cancer Pathways
Source: Cancer Res Commun. 2024 Dec 18;4(12):3151–64. doi: 10.1158/2767-9764.CRC-24-0285 (PMC11652962; doi:10.1158/2767-9764.CRC-24-0285)
Supplement: Table S6 — Benchmarking results for breast cancer subtype classification with cross-validation [file crc-24-0285_table_s6_suppst6.docx]

## Table S6 Benchmarking results for breast cancer subtype classification with cross-validation

|  | **Accuracy mean**  **± 95%CI** | **Macro-average F1-score mean**  **± 95%CI** | **AUROC mean**  **± 95%CI** | **Stability** |
| --- | --- | --- | --- | --- |
| **DeePathNet** | **0.902 ± 0.0081** | **0.868 ± 0.0115** | **0.980 ± 0.0030** | **0.019** |
| Random forest (RF) | 0.844 ± 0.0095 | 0.672 ± 0.0178 | 0.969 ± 0.0038 | 0.026 |
| *k*-NN | 0.816 ± 0.0097 | 0.697 ± 0.0200 | 0.897 ± 0.0094 | 0.033 |
| PCA+RF | 0.696 ± 0.0109 | 0.429 ± 0.0111 | 0.880 ± 0.0094 | 0.027 |
| mixOmics | 0.749 ± 0.0525 | 0.676 ± 0.0369 | 0.731 ± 0.0169 | 0.090 |
| moCluster+RF | 0.751 ± 0.0090 | 0.492 ± 0.0152 | 0.917 ± 0.0051 | 0.025 |
| MOVE+RF | 0.763 ± 0.0099 | 0.510 ± 0.0161 | 0.914 ± 0.0094 | 0.030 |
| scVAEIT+RF | 0.818 ± 0.0078 | 0.743 ± 0.0137 | 0.949 ± 0.0052 | 0.023 |

Benchmarking six methods to predict breast cancer subtypes by reporting cross-validation performance. Cells in bold represent the best performance.
