# Supplementary material for: DeePathNet: A Transformer-Based Deep Learning Model Integrating Multiomic Data with Cancer Pathways
Source: Cancer Res Commun. 2024 Dec 18;4(12):3151–64. doi: 10.1158/2767-9764.CRC-24-0285 (PMC11652962; doi:10.1158/2767-9764.CRC-24-0285)
Supplement: Table S7 — Generalization errors for breast cancer subtype classification [file crc-24-0285_table_s7_suppst7.docx]

## Table S7 Generalization errors for breast cancer subtype classification

|  | **Accuracy** | **Macro-average**  **F1-score** | **AUROC** |  |
| --- | --- | --- | --- | --- |
| **DeePathNet** | **0.902** | **0.798** | **0.971** |  |
| Random forest | 0.295 | 0.263 | 0.694 |  |

Comparing two methods by evaluating generalization errors of breast cancer subtype prediction. Cells in bold represent the best performance.
