# Supplementary material for: DeePathNet: A Transformer-Based Deep Learning Model Integrating Multiomic Data with Cancer Pathways
Source: Cancer Res Commun. 2024 Dec 18;4(12):3151–64. doi: 10.1158/2767-9764.CRC-24-0285 (PMC11652962; doi:10.1158/2767-9764.CRC-24-0285)
Supplement: Table S8 — Ablation study of DeePathNet on drug response prediction [file crc-24-0285_table_s8_suppst8.docx]

## Table S8 Ablation study of DeePathNet on drug response prediction

|  | **R^2^ mean ± 95%CI** | **MAE mean ± 95%CI** | **Pearson's r mean ± 95%CI** |
| --- | --- | --- | --- |
| **DeePathNet** | 0.208 ± 0.0118 | 0.933 ± 0.0274 | **0.476 ± 0.0119** |
| **DeePathNet (Transformer-only)** | **0.214 ± 0.0111** | **0.928 ± 0.027** | 0.475 ± 0.0116 |
| MLP | -0.674 ± 0.1632 | 1.305 ± 0.047 | 0.391 ± 0.0159 |
| Random forest | 0.027 ± 0.0200 | 1.07 ± 0.0315 | 0.390 ± 0.0143 |

Comparing DeePathNet performance using randomly wired pathways and plain neural network on drug response. Cells in bold represent the best performance.
