# Supplementary material for: DeePathNet: A Transformer-Based Deep Learning Model Integrating Multiomic Data with Cancer Pathways
Source: Cancer Res Commun. 2024 Dec 18;4(12):3151–64. doi: 10.1158/2767-9764.CRC-24-0285 (PMC11652962; doi:10.1158/2767-9764.CRC-24-0285)
Supplement: Table S9 — Ablation study of DeePathNet on cancer type and breast cancer subtype classification [file crc-24-0285_table_s9_suppst9.docx]

## Table S9 Ablation study of DeePathNet on cancer type and breast cancer subtype classification

|  | **Accuracy** | **Macro-average**  **F1-score** | **AUROC** |  |
| --- | --- | --- | --- | --- |
| ***Cancer type*** | |  |  |  |
| **DeePathNet** | **0.963** | 0.935 | **0.998** |  |
| DeePathNet (Transformer-only) | **0.963** | **0.936** | **0.998** |  |
| MLP | 0.855 | 0.763 | 0.967 |  |
| Random forest | 0.951 | 0.895 | 0.997 |  |
|  | |  |  |  |
| ***Breast cancer subtype*** | |  |  |  |
| **DeePathNet** | **0.902** | **0.798** | **0.971** |  |
| DeePathNet (Transformer-only) | 0.802 | 0.644 | 0.961 |  |
| MLP | 0.459 | 0.369 | 0.858 |  |
| Random forest | 0.295 | 0.263 | 0.694 |  |

Comparing DeePathNet performance using a Transformer-only model with randomly wired pathways and plain neural network on cancer type and breast cancer subtype classification. The random wired pathways were repeated ten times. Mean values with 95% confidence intervals are reported.
