# Supplementary material for: DeePathNet: A Transformer-Based Deep Learning Model Integrating Multiomic Data with Cancer Pathways
Source: Cancer Res Commun. 2024 Dec 18;4(12):3151–64. doi: 10.1158/2767-9764.CRC-24-0285 (PMC11652962; doi:10.1158/2767-9764.CRC-24-0285)
Supplement: Materials and Methods — Evaluation metrics [file crc-24-0285_materials_and_methods_suppsmm.docx]

# Supplementary Materials and Methods

## Evaluation metrics

For regression, R^2^, MAE and Pearson’s *r* were used to evaluate the performance and they are defined as follows:

$$R^{2}=1-\frac{\sum_{i=1}^{n} \left( y_{i}-\hat{y}_{i} \right)^{2}}{\sum_{i=1}^{n} \left( y_{i}-\bar{y} \right)^{2}}$$

$$MAE=\frac{\sum_{i=1}^{n} \left| \left( y_{i}-\hat{y}_{i} \right) \right|}{n}$$

$$Pearson^{'}s r=\frac{\sum_{i=1}^{n} \left( y_{i}-\bar{y} \right)(\hat{y}_{i}-\bar{\hat{y}})}{\sqrt{\sum_{i=1}^{n} \left( y_{i}-\bar{y} \right)^{2}}\sqrt{\sum_{i=1}^{n} \left( \hat{y}_{i}-\bar{\hat{y}} \right)^{2}}}$$

For a given drug, $y_{i}$ represents the actual IC_50_ of cell line $i$, $\hat{y}_{i}$ represents the predicted IC_50_ value of cell line $i$, $\bar{y}$ represents the mean value of all actual IC_50_ values, $\bar{\hat{y}}$ represents the mean value of all predicted IC_50_ values, and $n$ represents the total number of cell lines. For classification, multiple metrics were used to evaluate the predictive performance of DeePathNet and other models, including accuracy, macro-average F_1_-score, precision, recall, AUROC, AUPRC and stability. Let TP, TN, FP, FN represent true positive, true negative, false positive and false negative predictions. Accuracy is defined as $\frac{TP+TN}{TP+TN+FP+FN}$. Precision is defined as $\frac{TP}{TP+FP}$. Recall is defined as $\frac{TP}{TP+FN}$. Then the F_1_-score is calculated as the harmonic mean of the precision and recall, defined as $\frac{2\cdot Precision\cdot Recall}{Precision+Recall}$. The macro-average F_1_-score is calculated by computing the arithmetic mean of F_1_-scores from all the cancer types or subtypes. The ROC curve is created by plotting the recall and false positive rate ($\frac{FP}{FP+TN}$) at various thresholds. AUROC is calculated as the area under the ROC curve. The precision and recall (PR) curve is created by plotting the precision and recall at various thresholds, and the AUPRC is calculated as the area under the PR curve. The stability is measured by the standard deviation.
